# Multicomponent Vaccines against Group A Streptococcus Can Effectively Target Broad Disease Presentations

**DOI:** 10.3390/vaccines9091025

**Published:** 2021-09-15

**Authors:** Helen A. Shaw, James Ozanne, Keira Burns, Fatme Mawas

**Affiliations:** The National Institute of Biological Standards and Control (NIBSC), Blanche Lane, South Mimms, Potters Bar, London EN6 3QG, UK; james.ozanne@nibsc.org (J.O.); keira.burns@nibsc.org (K.B.); Fatme.Mawas@nibsc.org (F.M.)

**Keywords:** Group A Streptococcus, *Streptococcus pyogenes*, vaccine, opsonophagocytosis, IL-8, IgG, SpyCEP, Cpa, Mac-1 IdeS, MalE

## Abstract

Group A Streptococcus (GAS) is an important global human pathogen, with a wide range of disease presentations, from mild mucosal infections like pharyngitis to invasive diseases such as toxic shock syndrome. The effect on health and mortality from GAS infections is substantial worldwide, particularly from autoimmune sequelae-like rheumatic heart disease (RHD), and there is currently no licenced vaccine. We investigated protein antigens targeting a broad range of GAS disease presentations as vaccine components in individual and combination formulations. The potency and functional immunity generated were evaluated and compared between groups. Antibodies against all components were found in pooled human IgG (IVIG) and an immune response generated following the subcutaneous immunisation of mice. A combination immunisation showed a reduction in IgG response for SpyCEP but an increase for Cpa and Mac-1 (IdeS). An opsonophagocytosis assay (OPA) showed the killing of GAS with immune sera against M protein and combination groups, with a lower killing activity observed for immune sera against other individual antigens. Specific antigen assays showed functional immunity against SpyCEP and Mac-1 from both individual and combination immunisations, with the activity correlating with antibody titres. However, efficient blocking of the binding activity of Cpa to collagen I and fibronectin could not be demonstrated with immune sera or purified IgG. Our data indicate that combination immunisations, while effective at covering a broader range of virulence factors, can also affect the immune response generated. Further, our results showed that an OPA alone is inadequate for understanding protection from vaccination, particularly when considering protection from immune evasion factors and evaluation of the colonisation leading to pharyngitis.

## 1. Introduction

Group A Streptococcus (GAS) is a global bacterial pathogen causing a wide range of disease presentations, from mild pharyngitis to potentially fatal invasive and toxin-mediated diseases. Autoimmune sequelae can occur following mild infections, leading to rheumatic heart disease (RHD), amongst other conditions, which is a global healthcare burden, leading to a significant loss of disability-adjusted life years (DALYs) [1]. While antibiotic treatment has proven effective in high-income countries at reducing the prevalence of RHD, it is still endemic in low- and middle-income countries (LMIC) [1], such that the WHO have highlighted the important need for a global vaccine against GAS [2].

Currently, there is no licenced vaccine to prevent GAS infection, with few candidates undergoing clinical trials [3,4]. These most progressed vaccines target the M-protein, either as a multivalent formulation against the variable N-terminal domain [3] or against the conserved C-terminal domain [4]. The multivalent M-protein vaccine has been proven to provide effective protection in animal models but is limited by low serotype coverage against those circulating in LMICs, with the further risk of the emergence of nonvaccine serotypes [5]. Vaccines targeting the conserved region have shown some protection in animal models but have a reduced ability for opsonisation [6]. Several multicomponent vaccines have been tested preclinically but have yet to enter clinical trials [7,8,9]. These have focussed on targets identified through reverse vaccinology, combining the sequence conservation, localisation and immunogenicity of conserved antigens, as shown with the *Streptococcus pyogenes* cell envelope protein (SpyCEP), *S. pyogenes* Adhesion and Division protein (SpyAD) and streptolysin O (SLO) [7] or by identifying antigens generating an immune response through natural infections from pooled human IgG (Spy7) [8]. Other multicomponent vaccine studies have combined individual promising candidates such as SLO, SpyCEP, C5a peptidase (SCPA), arginine deiminase (ADI) and trigger factor (TF) (Combo5) [6]. The WHO-preferred product characteristics are the prevention of colonisation (pharyngeal or skin) as a predeterminant of the prevention of acute rheumatic fever leading to RHD; however, based on the rates of pharyngitis and systemic infection in adults [10], an immunity to pharyngitis does not necessarily protect against invasive disease. Therefore, it is important for the scope of a multicomponent vaccine to cover both colonisation and invasive disease virulence factors whilst avoiding epitopes that could lead to the induction of autoimmune responses [11,12]. It is also imperative that relevant or suitable functional assays are established to demonstrate this breadth of protection. The road to a vaccine has been hindered by a lack of immunoassays, absence of correlates of protection and limited surrogates of protection. Although opsonophagocytosis assays are being established, functional killing does not correlate with protection for all promising vaccine candidates, such as SpyCEP [9,13,14,15].

In the present study, we have prepared and evaluated four recombinant protein antigens, in addition to M-proteins, particularly to be representative of different stages of GAS infection: Cpa, a pilin tip protein which caps the pilus polymer has been shown to display adhesion properties in some serotypes, hypothesised to play a role in GAS colonisation [16,17]; MalE, a cell associated maltodextrin binding protein essential for GAS survival in saliva [18,19]; Mac-1/IdeS (henceforth referred to as Mac), a secreted IgG protease that plays a role in the evasion of immune mechanisms by preventing opsonisation and phagocytosis [20,21,22] and SpyCEP, a cell surface and secreted IL-8 protease that reduces the recruitment of neutrophils, allowing GAS to evade immune clearance [23,24] and additionally playing a role in the invasion of cells and dissemination of GAS [25,26]. SpyCEP is included in the GSK combo vaccine and Combo5 and has been shown to be protective against infection in several preclinical studies [25,26], while the remaining three antigens are novel to vaccine evaluations.

The main aim of our study was to evaluate the immunogenicity of these antigens individually and assess the effect of combining antigens on the level and functionality of the antibody response. In addition, we explored different laboratory methods or in vitro assays to understand the correlates of protection generated from immunisation with the different vaccine candidate antigens.

## 2. Materials and Methods

### 2.1. Bacterial Growth Conditions

*Escherichia coli* were grown in Luria Broth (LB) or LB agar supplemented with 30-μg/mL kanamycin where appropriate for the cloning of recombinant antigens. For the production of recombinant proteins, Overnight Express Instant TB media (Merck, Darmstadt, Germany) was used with kanamycin. GAS M1 (NCTC 8198) was routinely grown in THY media (Todd Hewitt with 0.2% yeast extract) or on TH agar plates at 37 °C in 5% CO_2_. To prepare single-use frozen GAS cultures for use in functional assays, bacteria were grown in THY broth to mid-log (~OD 0.6). A 10-mL culture was mixed with 10-mL fresh THY and 8-mL 70% glycerol. Five hundred microlitres was transferred to individual 1.5-mL tubes and stored at −80 °C.

### 2.2. Preparation of Recombinant GAS Antigens

Gblocks (IDT) and GeneArt Strings (Thermo Fisher Scientific, Waltham, MA, USA) were synthesised from GAS antigens from the SF370 genome, as indicated in Supplementary Figure S1, without N-terminal signal peptides or C-terminal domains for cell wall associations. The antigens were cloned as full-length constructs, except for SpyCEP, which was cloned as a catalytically inactive fragment [27] with residue 1317 mutated C to T to remove an internal NcoI site. Gene fragments were digested with NcoI and XhoI for ligation into pET28a to generate a C-terminal 6XHIS tag and transformed into NEB5α (NEB) *E. coli*. Following the Sanger sequencing confirmation of cloned constructs, plasmids were transformed into BL21 (DE3) *E. coli*, and the cultures were grown in Overnight Express Instant TB media for the induction of protein expression. Fifty millilitres of culture pellets were freeze-thawed and lysed by suspension in lysis buffer (1× Bug Buster (Merck, Darmstadt, Germany), 50-mM Tris-HCl, pH 8.0, 500-mM NaCl, 1-mg/mL lysozyme and 40-μg/mL DNase) 20 min at RT with rotation, followed by 30 min at 37 °C. Suspensions were harvested by centrifugation for 10 min at 17,000× *g* and supernatants filtered through a 0.2-μm filter before loading onto a 1-mL HisTrap HP column (Cytiva life sciences, Malborough, MA, USA). The column was washed with 50-mM Tris-HCl, pH 8.0, 500-mM NaCl, 20-mM imidazole and recombinant protein eluted with 50-mM Tris-HCl, pH 8.0, 500-mM NaCl and 250-mM imidazole. Fractions containing recombinant protein were pooled and further purified by size exclusion chromatography on a 16.60 Sephacryl S 300 gel filtration column (Cytiva life sciences, Malborough, MA, USA) in 50-mM Tris-HCl, pH 7.5 and 150-mM NaCl. High-purity fractions were pooled and concentrated by spin columns at 3 K MWCO (Merck) and endotoxin removed using the Pierce High-Capacity Endotoxin Removal Resin (Thermo Fisher Scientific, Waltham, Massachusetts, USA). Purified recombinant proteins were tested for purity and analysed for identity by mass spectrometry and a LAL test performed for endotoxin levels <24 IU/mL.

### 2.3. Animals and Immunisations

Groups of female 6-8-week-old BALB/c mice (Charles River Laboratories, Saffron Walden, UK) were immunised subcutaneously with 20 μg of recombinant protein antigens individually (5 mice) or in combination with 20 μg of each antigen (10 mice) in the presence of aluminium hydroxide (Alum) adjuvant (100 μg/dose). Antigens were adsorbed to Alum for 90 min at RT prior to immunisations. For combination groups, antigens were combined and then adsorbed into Alum in a single incubation. Each mouse received 3 doses at 14-day intervals. Serum was collected prior to immunisation and terminal bleeds collected two weeks after the final immunisation. Animal studies were conducted according to the UK Home Office regulations and were approved by the local ethics committee.

### 2.4. Purification of IgG from Mouse Sera

A Mouse Antibody purification kit (Ab128745, Abcam, Cambridge, UK) was used to purify IgG from immune sera. Briefly, 0.3-mL wash buffer was added to each vial of mouse resin and mixed by inversion before transfer to a spin cartridge and spun for 30 s at 17,000× *g*. A 10× binding buffer was added to each sera sample to a maximum volume of 500 μL, transferred to the spin cartridge, capped and incubated 3 h at RT with rotation. The samples were spun for 30 s at 17,000× *g* and washed with 0.5-mL wash buffer four times. In a clean collecting tube, the IgG antibodies were eluted by incubation with 100-μL elution buffer for 2 min RT with rotation before spinning 30 s at 17,000× *g*. The samples were neutralised with the addition of 25-μL neutralisation buffer. The elution was conducted four times, and the elutions with the highest protein concentration were pooled.

### 2.5. ELISA to Measure IgG Antibody Titre

MaxiSORP 96-well plates (Nunc, Roskilde, Denmark) were coated with 50 μL/well of 0.5-μg/mL recombinant protein and incubated overnight at 4 °C. Plates were washed 7 times with PBS 0.05% Tween-20 (PBS-T) and blocked with 100 μL of 1% bovine serum albumin (BSA) 0.3% Tween-20 (B-PBS-T) at 37 °C for 1 h. One hundred microlitres of serum dilutions in B-PBS-T were added to the plates and incubated at 37 °C for 2 h. The plates were washed 7 times and 100-μL anti-mouse IgG-HRP (Sigma, St. Louis, MO, USA, A9044, diluted 1/20,000), or anti-human IgG-HRP (Sigma, St. Louis, MO, USA, A8792, diluted 1/5000) was added to the plates and incubated at 37 °C for 1 h. The plates were washed 7 times, and 100-μL TMB substrate (3,3′,5,5′-tetramethylbenzidine, Thermo Fisher Scientific, Waltham, MA, USA) was added and incubated at RT for 15 min, and the reaction was stopped with 100 μL of 1-M sulphuric acid. The plates were read at 450 nm on a SoftMax Pro plate reader. The antibody titre was determined as the serum dilution giving an OD 0.5 using the interpolation of a sigmoidal curve in GraphPad Prism.

### 2.6. Fractionation of GAS Cultures for Protein Analysis

Overnight cultures of GAS were subcultured to OD 0.05 in THY media and grown to the mid-log phase (OD~0.7). Cells were harvested at 4000× *g* and resuspended in a cell wall extraction buffer (10-mM Tris-HCl, pH 8.0, 30% (*w/v*) raffinose, 100-U/mL mutanolysin, 1-mg/mL lysozyme and 20-U/mL PlyC with protease inhibitor cocktail) to OD 20. Suspensions were incubated with agitation for 3 h at 37 °C before harvesting at 6000× *g* 20 min at RT. Culture supernatants were concentrated by filtering through a 0.2-μm filter and precipitating proteins with 10% TCA. The supernatants were incubated for 18 h on ice before harvesting at 17,000× *g* for 20 min. Precipitated proteins were washed three times with 90% acetone before air drying and resuspended in 10-mM Tris-HCl, pH 8.0, to OD 20.

### 2.7. Visualisation of Protein Antigens

One microgram of protein antigen or GAS cellular fraction was loaded onto Bolt 4–12% Bis-Tris gels (Thermo Fisher Scientific, Waltham, MA, USA) and separated by PAGE. Proteins were either stained by Coomassie Brilliant Blue or transferred onto nitrocellulose membranes for analysis by Western blots. Western blots were blocked for 1 h with 3% milk in PBS before incubation with mouse immune sera (1/500 Cpa, MalE; 1/1000 PI, SpyCEP, Mac, ALL; 1/3000 M1 and M3) for 1 h in 3% milk PBS-T (0.05% Tween-20). Following washes in PBS-T, HRP-conjugated rabbit anti-mouse IgG (Sigma, St. Louis, MO, USA, A9044) secondary antibody were added 1/5000 in 3% milk PBS-T for 30 min. Western blots were detected with Pierce ECL Western Blotting Substrate (Thermo Fisher Scientific, Waltham, MA, USA).

### 2.8. Immunofluorescence Staining and Flow Cytometry Analysis

Cultures of GAS were grown to log-phase OD 0.4–0.8 and harvested for 5 min at 4000× *g*. The bacteria were suspended in PBS with 10% goat serum and incubated for 20 min at RT. A 1-mL suspension per test condition was harvested before incubation in 100-μL staining buffer (0.1% BSA and 10% goat serum PBS) with and without 1:4 dilution of immune mouse serum for 1 h at 4 °C. Cells were washed with 0.1% BSA-PBS before incubation in 100-μL goat-anti-mouse-FITC (Thermo Fisher Scientific, Waltham, MA, USA, A16067) 1:3000 in 0.1% BSA-PBS for 45 min at 4 °C. Cells were washed with 0.1% BSA-PBS and suspended in 1 mL of fixative (2% formaldehyde and 50% PBS). Sample data was collected on a BD FACS Canto II flow cytometer, with further analyses carried out using FlowJo Software.

### 2.9. Opsonophagocytosis Assay (OPA)

HL60 cells (ATCC CCL-240) were grown routinely in RPMI, 10% foetal calf serum (FCS), 1% glutamine and 1% penicillin–streptomycin and differentiated with 0.8% DMF at 4 × 10^5^ cells/mL in 100 mL for 5 to 6 days. Frozen GAS cultures were tested with differentiated HL60 cells and 2% baby rabbit complement (Pel-Freez Biologicals) or heat inactivated complement at a bacteria:cell ratio of 1:400 with <35% nonspecific killing [13]. To perform the OPA, GAS frozen cultures were thawed, washed and diluted to the required input dilution in OBB (1 × HBSS with salts, 0.1% gelatin and 5% FCS). For opsonisation, 10 μL of diluted GAS was incubated with 20 μL of immune and nonimmune sera dilutions or OBB in 96-well round-bottom plates with shaking at 700 rpm for 30 min at RT. HL60 cells were harvested at 300× *g* for 5 min and washed sequentially in 1 × HBSS without salts and 1 × HBSS with salts before resuspension in OBB to 1 × 10^7^ cells/mL. HL60s were mixed at a 4:1 ratio with 2% complement or heat-inactivated complement, and 50 μL were added to opsonised GAS before incubation for 90 min at 37 °C of 5% CO_2_ with shaking at 200 rpm. Following that, the incubation plates were placed on ice for 20 min to stop phagocytosis. The cultures were resuspended and spot-plated on THY agar for CFU enumeration.

### 2.10. IL-8 Cleavage Assay

An IL-8 DuoSET ELISA kit (RnD, DY208-05) was used to perform IL-8 cleavage assays as follows. GAS cultures were grown to mid-log (OD 0.6) in THY broth, harvested at 4000× *g* and supernatants 0.2-μm filter sterilised. Supernatants were then diluted 1/40 with 125-pg/mL IL-8 (RnD, 208-IL-010/CF) in a total volume of 100 μL and incubated at 37 °C for 16 h. Where necessary, the supernatants were preincubated with mouse sera dilutions at 37 °C for 1 h prior to IL-8 addition. MaxiSORP 96-well plates (Nunc, Roskilde, Denmark) were coated with 100 μL of 4-μg/mL capture antibody at RT overnight. The plates were blocked with 300-μL 1% BSA, 1 × PBS and 0.05% Tween-20 at RT for 1 h before the addition of 100-μL supernatants and incubation at RT for 2 h. A 100-μL detection antibody was added and incubated at RT for 2 h, and 100-μL streptavidin was added at RT for 20 min before detection with 100-μL TMB. The reactions were stopped with 100 μL of 1-M sulphuric acid, and absorbance was read at 450 nm. All steps barring the addition of sulphuric acid were separated by 7 washes with PBS-T (0.05%).

### 2.11. IgG Cleavage Assay

Ten nanograms of recombinant Mac was incubated with 100-ng human IgG (Sigma, St. Louis, MO, USA, I2511) in a total volume of 10 μL of 1% BSA PBS-T (0.03% Tween-20) and incubated at 37 °C for 60 min. Where necessary, the Mac was preincubated with mouse sera dilutions at 37 °C for 45 min. The samples were run on a 4–12% Tris-BIS Novex gel and transferred to a nitrocellulose membrane. The membranes were probed with 1/5000 HRP-conjugated rabbit anti-human IgG (Sigma, St. Louis, MO, USA, A8792) and detected with the Pierce ECL Western Blotting Substrate (Thermo Fisher Scientific, Waltham, MA, USA).

### 2.12. Binding Assays

MaxiSORP 96-well plates were coated with 50 μL of 2.5-μg (50 μg/mL) collagen I (Sigma, St. Louis, MO, USA, C7774) or Fibronectin (Sigma, St. Louis, MO, USA, F2006) overnight at 4 °C. The plates were blocked with 1% BSA PBS-T (0.05% Tween-20) for 1 h at 37 °C before the addition of 100 μL of 80-pmol Cpa to assay the wells and static incubation for 1 h at 37 °C. After washing with PBS-T seven times, the plates were incubated with 100 μL of 1/2000 anti-HIS-HRP-conjugated antibody (Ab1187, Abcam) for 1 h at 37 °C. After seven washes with PBS-T, 100 μL of TMB (Thermo Fisher Scientific, Waltham, MA, USA) was added to the plates for 15 min at RT before neutralisation with an equal volume of 1-M sulphuric acid, and the absorbance was read at 450 nm. For the blocking of binding, Cpa was incubated with immune sera or purified IgGs for 1 h at 37 °C with rotation before being added to the plates, as described above.

## 3. Results

### 3.1. Protein Production and Characterisation

**Figure 1:**
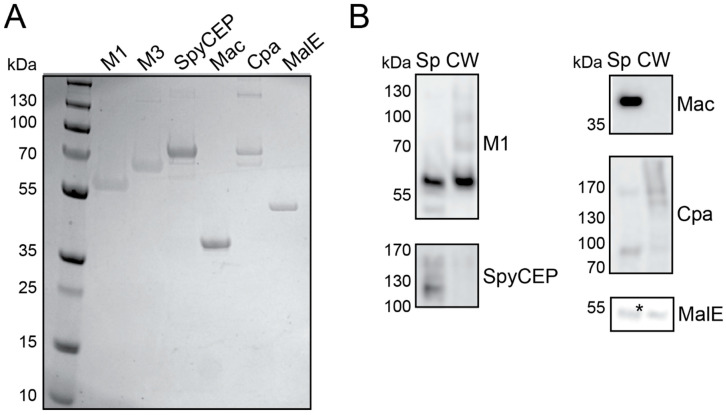
Recombinant vaccine antigens induced a specific antibody response capable of recognising the native proteins. Recombinant proteins were purified from *E. coli* to a high purity and generated a specific antibody response to each antigen, which could recognise native proteins from the GAS cell extracts. (**A**) Coomassie-stained SDS-PAGE of 1 μg of each recombinant protein antigen, as indicated. (**B**) Concentrated supernatants (Sp) and cell wall (CW) extracts from an M1 culture were separated by SDS-PAGE and probed with antigen-specific immune sera, as indicated. Mouse anti-M1, SpyCEP and Mac: 1:1000 and Cpa and MalE: 1:500. Secondary antibody, rabbit anti-mouse-HRP: 1:1000. * Nonspecific band. Full-length Western blots alongside representative M1 group pre-immune detection are shown in Supplementary Figure S2.

GAS protein antigens SpyCEP, Mac-1 (IdeS), Cpa and MalE along with M-proteins were purified from *E. coli* to a high purity and yield (Table 1 and Figure 1A). SpyCEP, Mac and MalE are highly conserved antigens between GAS serotypes, while Cpa shows serotype variations. Conserved fibronectin-binding proteins were not successfully expressed and purified within the confines of the project to represent the colonisation factors. The well-characterised M1 and M3 proteins were included as control antigens due to their well-characterised protective properties, function in immunoassays and serotype variations. To confirm the importance of a response to these antigens from natural human infections in protection, the presence of antibodies against these antigens in human pooled IgG (IVIG) was analysed and demonstrated the presence of antibodies to the selected GAS protein antigens, with high antibody levels against the M protein, SpyCEP and Mac but lower titres for MalE and Cpa (Table 1).

Mouse immune sera raised against recombinant antigens were tested for the recognition of native GAS proteins. Culture supernatants and cell wall extracts from late exponential-phase M1 GAS cultures were probed by Western blotting with antigen-specific immune sera to demonstrate the detection of native proteins (Figure 1B). All antigens could be detected with specific immune sera at their predicted molecular weights in secreted or cell surface locations.

### 3.2. Immunisation with Recombinant Protein Antigens Induces a High IgG Antibody Response for Most Candidates

To assess the IgG response to immunisation with the GAS antigens, BALB/c mice were immunised subcutaneously with 20 μg of each individual candidate antigen in the presence of Alum. The IgG antibody for each protein antigen was quantified by ELISA, with the data presented as the geometric mean titre (GMT) for each group in Table 2. A high immunogenicity was observed for M1, M3 and SpyCEP as individual components (GMT: 34,542, 97,715 and 125,934, respectively). An intermediate response was observed for Cpa (2870) and Mac (4856), with a low response to MalE (93), which was not significantly above the pre-immune level.

### 3.3. Immunisation in Combination Can Alter the Immune Response to Protein Antigens

A combination formulation with all antigens “ALL” or all antigens without M proteins “NoM” containing 20 μg of each antigen was used to immunise BALB/c mice to investigate any effects on the IgG response against each antigen from interactions in a multicomponent vaccine (Table 2). When the antigens were combined, the M1 and M3 responses remained consistent with the individual immunisation titres. The response to SpyCEP showed a significant four-fold decrease in titre (*p* < 0.01) in the “ALL” combination group, but when the M proteins were excluded from the formulation, the SpyCEP response returned to the high level observed for individual immunisation (GMT: 115,946 and 125,934, respectively). There was a small, but statistically significant, two-fold increase in the Mac antibody titre in the “ALL” combination group compared to the individual immunisation (*p* < 0.05) but not in the “NoM” group. The response to Cpa showed an 18-fold increase in the titre (*p* < 001) in the “ALL” combination group but not in the “NoM” group, which was not significantly higher from the individual immunisation due to the wide variations in the response between individual mice. The IgG response to MalE showed a significant increase in the titre in the combination group compared with individual immunisation (268; *p* < 0.05), but this response was not significantly above the baseline signal from nonimmune sera.

### 3.4. Antibodies Bind the Surface of GAS and Demonstrate Functional Killing Activity

**Figure 2:**
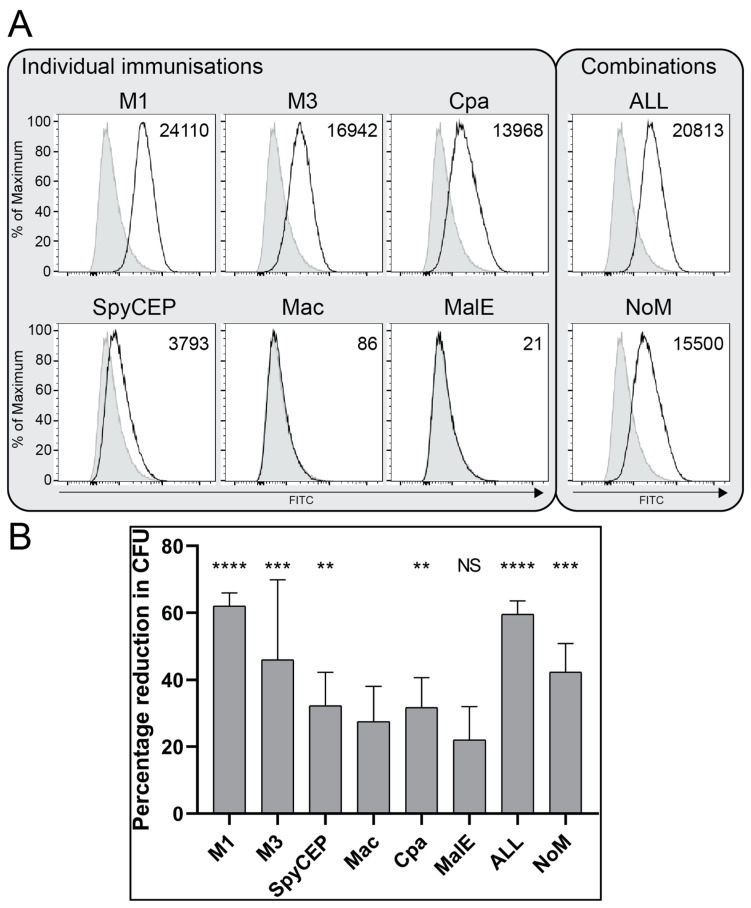
Vaccine antigens produce antibodies that bind the surface of GAS and target GAS for killing by neutrophils. Mouse antibodies raised by immunisation were tested for binding to NCTC 8198 GAS cells by immunostaining with an analysis by flow cytometry (**A**), and the killing activity in a HL60-based opsonophagocytosis assay (OPA) (**B**). (**A**) Flow cytometry analysis of GAS M1 cells immunostained with a 1:4 dilution of mouse sera against individual or combination antigens compared with nonimmune sera. Specific binding is shown by a shift in the FITC fluorescence. Pale grey, normal mouse sera negative control; solid line, indicated immune sera. Numbers indicate a population comparison *T(X)*, with a *T(X)* value >200 considered significantly above the controls calculated by the FlowJo probability binning algorithm. Further negative controls are shown in Supplementary Figure S2. The arrow indicates an increasing FITC fluorescence. (**B**) OPAs were conducted with DMF-differentiated HL60 cells and a baby rabbit complement. The killing of GAS cells with immune mouse sera was quantified by CFU relative to the controls containing nonimmune sera. One-way ANOVA comparison with nonimmune sera is indicated: NS, not significant. ** *p* < 0.01; *** *p* < 0.001 and **** *p* < 0.0001.

To evaluate the binding of antibodies generated from the immunisation experiments to the surface of M1 GAS cells, immunofluorescent staining and a flow cytometry analysis using immune and nonimmune mouse sera were performed. The analysis showed binding antibodies for the M proteins and cell=associated Cpa (Figure 2A). SpyCEP is both a cell wall protein and cleaved into the supernatant, and the results, therefore, showed a small shift in fluorescence intensity above the control baseline, while the antibodies to the secreted protein Mac showed no binding. MalE with no discernible immune response demonstrated no binding antibodies. The immune sera from both combination groups ALL and NoM showed binding to GAS, but the inclusion of M proteins showed more of a shift in the fluorescence intensity from the controls (*T*(*X*) 20,813 and 15,500, respectively). Interestingly there was a slight reduction in the staining when all the antigens were included compared to M1 alone (*T*(*X*) 20,813 and 24,110, respectively).

Functional protective antibodies have classically been demonstrated as killing a target bacterium in the presence of a complement and neutrophils. Immune sera from mice immunised with individual antigens or a combination were investigated for a reduction in the number of colony-forming units (CFU) as a measure of the killing activity compared with nonimmune normal mouse sera. As expected, M1 immune sera was able to effectively kill M1 GAS cells in the OPA compared with nonimmune sera (*p* < 0.0001), while sera raised against the M3 protein showed less activity with a high variance (*p* < 0.001), demonstrating serotype specificity (Figure 2B). Immune sera against the remaining antigens SpyCEP, Mac, Cpa and MalE showed negligible killing activity (*p* < 0.01 for SpyCEP and Cpa only). The combination immunisation with all antigens showed significantly reduced CFU compared to the nonimmune sera (ALL *p* < 0.0001 and NoM *p* < 0.001).

### 3.5. Immune Sera Can Block the Functional Activity of Secreted GAS Virulence Factors

Several prospective antigens have been shown to be protective and effective vaccine candidates despite low OPA titres, such as SpyCEP [15,24]. SpyCEP and Mac immune sera are unlikely to show protective activity in an assay such as an OPA due to their localisation and the functional protease activity of the antigens. Therefore, to assess whether neutralising antibodies were present, antigen-specific assays were conducted.

**Figure 3:**
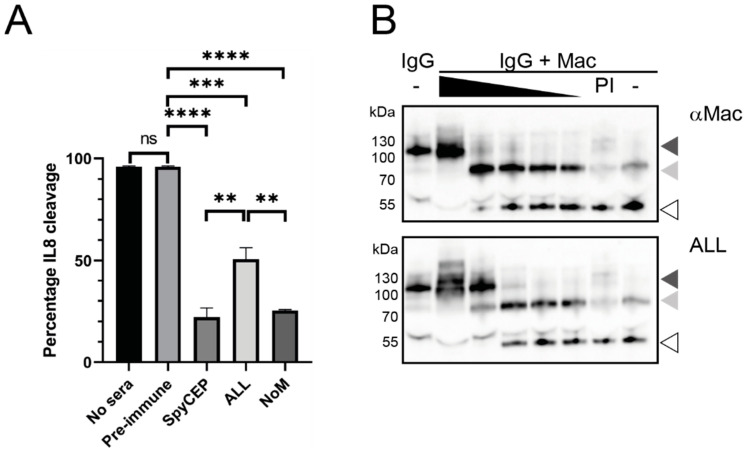
Immune sera can neutralise the enzymatic activity of GAS virulence factors. SpyCEP and Mac (IdeS) are two proteases secreted by GAS that aid in immune evasion. SpyCEP cleaves IL-8, and Mac cleaves IgG. The activity of the enzymes was assessed in the absence of sera, pre-immune sera (PI) or immune sera from individual antigen groups and combination groups. (**A**) The cleavage of IL-8 by SpyCEP in the absence of immune sera, with significant neutralisation upon 1:1 incubation with immune mouse sera, as detected by ELISA. Ns, not significant; ** *p* < 0.01, *** *p* < 0.001 and **** *p* < 0.0001 (one-way ANOVA). (**B**) The cleavage of human IgG by Mac is neutralised by the titration of immune sera. IgG or IgG + Mac were incubated in the absence of sera (-), immune sera titrations (black triangle, 10-fold titration from neat to 1:10,000) or pre-immune serum (PI). The cleavage reactions were separated by SDS-PAGE and human IgG detected by Western blot with rabbit anti-human IgG-HRP 1:5000. Dark-grey arrow, full-length IgG; light-grey arrow, partially cleaved IgG; white arrow, fully cleaved IgG. Full-length Western blot images are in Supplementary Figure S3.

The IL-8 cleavage activity by SpyCEP in GAS late-log supernatants was evaluated with and without immune sera. The incubation of GAS supernatants with IL-8 alone or in the presence of pre-immune serum resulted in a >95% cleavage of IL-8 (Figure 3A), with no significant difference between the controls. A 1:2 dilution of pooled immune sera from SpyCEP immunisation or combination immunisations resulted in a significant reduction in IL-8 cleavage compared with nonimmune sera (SpyCEP 22% *p* < 0.0001, ALL 51% *p* < 0.001 and NoM 25% *p* < 0.0001), which equated to 78%, 49% and 75% inhibition of cleavage, respectively. A significant difference in the functional immunity between individual SpyCEP immunisation and the “ALL” combination immunisation was observed (*p* < 0.01) (Figure 3A), and this was similar to the difference seen in the antibody level between the groups as measured by ELISA (see above). Despite this reduction in neutralising activity, the “ALL” combination immunisation still resulted in significantly reduced IL-8 cleavage compared to the controls. The functional immunity was dose-dependent, as shown in the antibody titration data (Supplementary Figure S3).

Mac cleaves human IgG (huIgG) at the hinge region, preventing the antibody-mediated phagocytosis of GAS, hence aiding immune evasion. In a cleavage assay, huIgG was incubated with recombinant Mac, and a ~55-kDa cleavage product was observed (Figure 3B, final lane) compared to full-length IgG without Mac (first lane). Pooled immune mouse sera were titrated in 10-fold dilutions and tested for neutralising activity on Mac to prevent the cleavage of huIgG. As can be seen in Figure 3B, neat anti-Mac serum was able to completely inhibit the cleavage activity of Mac, with the titration showing a gradual increase in Mac activity, which did not return to the complete activity seen with the pre-immune (PI) serum or Mac in the presence of no sera, even at a 1:10,000 dilution. Similar results were observed with the immune serum from the combination “ALL” group, with an estimated 10-fold higher titre in the neutralising activity, as shown by the retention of a full-length IgG molecule at a 1:10 dilution. This increased neutralising activity was also observed with immune serum from the “NoM” group (Supplementary Figure S3). Pre-immune (PI) sera showed no neutralisation of the Mac activity.

### 3.6. IgGs Are Not Effective at Blocking the Binding Activity of Cpa to Fibronectin and Collagen

**Figure 4:**
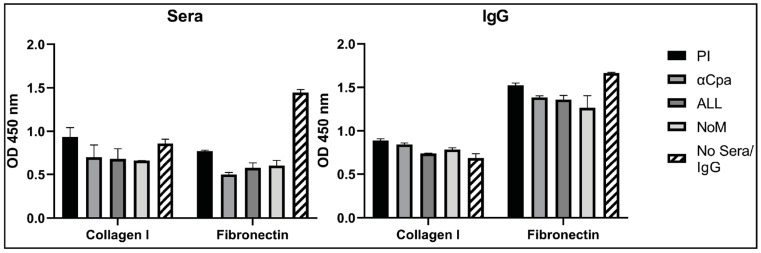
Specific blocking of Cpa binding to collagen I and fibronectin cannot be demonstrated with sera or purified IgG. Cpa shows binding to immobilized collagen I and fibronectin by ELISA. Binding was analysed in the presence and absence of sera or purified IgG. Bound Cpa was detected by rabbit anti-HIS-HRP 1:2000. PI, pre-immune.

Pili are involved in GAS colonisation potentially through interactions of the capping protein, Cpa, with extracellular matrix proteins. Cpa was demonstrated to bind to collagen I and fibronectin by ELISA (Figure 4, hatched bar). One in ten dilutions of nonimmune and immune sera were incubated with Cpa for 1 h prior to performing binding assays to determine whether the blocking of binding could be demonstrated. As Figure 4 shows, despite a small reduction in Cpa binding to collagen in the presence of immune sera from mice immunised with Cpa or a combination of antigens, this reduction was not statistically significant. Further, Cpa binding to fibronectin was significantly blocked with both immune and pre-immune (PI) sera (*p* < 0.0001), suggesting a nonspecific blocking ability from sera itself. Due to this, the IgGs were purified from all the groups to try to prevent the intrinsic blocking activity of sera. The experiments were repeated and again showed no significant blocking of the binding of Cpa to collagen I. There was a reduction in the binding to fibronectin for immune IgG compared with Cpa alone (αCpa *p* < 0.01, ALL *p* < 0.001 and NoM *p* < 0.0001). However, in real terms, this was not a clear blocking of binding, as it was a less-than 25% reduction in binding, with a nonsignificant reduction in binding when compared between nonimmune IgG (PI) and αCpa-immune sera. Similarly, the reduction in binding was less significant for the combination groups when compared with PI IgG (ALL *p* < 0.1, NoM *p* < 0.01).

## 4. Discussion

There is a high need for a vaccine against GAS globally, particularly to reduce the burden of RHD on DALYs and prevent mortality from invasive infections [2]. Due to broad GAS disease presentation and diverse serotype variations, particularly in Africa and the Pacific regions, finding an effective long-term vaccine strategy is challenging [11]. The M protein has been the focus of vaccine designs due to its dominant role in infections and effective serotype-specific protection. The inclusion of M1 and M3 proteins in our study validated the previous findings on serotype specificity and emphasised the important role the immunity to M proteins plays in the effective killing of GAS by opsonophagocytosis. Due to our constructs covering the full-length protein, both serotype-specific and conserved domains were present. This led to M3 antibodies recognising M1 GAS, as shown by the flow cytometry analysis, but this did not lead to the consistent effective killing of M1 cells in our OPA. Some killing activity can be connected by antibodies to the C-terminal conserved domain, as seen with J8 vaccines, but this is not as effective as antibodies to the variable serotype-specific N-terminus [32]. Serotype variations limit M protein use as a long-term vaccine strategy due to the risk of emergence and dominance of nonvaccine strains. Continued surveillance and refinement of the vaccine would be needed, as seen for pneumococcal vaccines [33]. Therefore, it has been of interest for the field to investigate the use of other candidates and their incorporation into multicomponent vaccines to achieve similar protection from infections by M protein immunity. Our combination formulation, which contained new antigens that have not been evaluated before (Mac, Cpa and MalE), showed encouraging data that, in the absence of M proteins’ effective killing, could be evident despite a low killing activity with antibodies against the individual antigens potentially through cumulative activity. Further, in this “NoM” group, the functional blocking of virulence factors with the induced antibodies was still as significantly effective as against the individual antigens. Further analyses should investigate the broad activity of this binding to GAS cells and killing activity across diverse serotypes.

SpyCEP has been demonstrated in numerous studies to be a protective antigen, but a killing assay does not correlate with this efficacy [11]. We corroborated these data, showing that, despite some binding to surface-exposed SpyCEP by antibodies through flow cytometry, there was not a high killing activity observed for anti-SpyCEP antibodies in an OPA. Immune sera to SpyCEP are, however, able to neutralise the SpyCEP cleavage of IL-8. This shows that an adaptive immune response to SpyCEP could neutralise GAS-mediated IL-8 cleavage, allowing for the recruitment of neutrophils to the site of infection and enabling innate immunity to contribute to the clearance of infection. In this study we showed that the presence of M proteins in the vaccine formulation reduced the immune response to SpyCEP. While a significant neutralisation of IL-8 cleavage was still observed, this was significantly less than the formulations without the M protein. It remains to be determined whether this was due to alterations in the structure of SpyCEP, a reduction in the adsorption to aluminium hydroxide (Al(OH)_3_) adjuvant due to steric hindrance or other factors. It is possible that, in our study, the adsorption of SpyCEP to Alum in the “ALL” group was not as good as in the SpyCEP alone group due to competition from the other antigens, especially the M proteins. Therefore, other adjuvants should be investigated to determine whether they will affect the response. It has previously been reported that the adjuvant selected can affect the protective effect from immunisation with GAS antigens, with Alum producing the least effective protection with the Combo5 vaccine in mice [15]. This was partially reflected in the antibody titres to antigens such as SpyCEP, though not to significant levels; however, there was a significant reduction in the SpyCEP IgG2c titre in the Alum group compared with the SMQ adjuvant. The M1 vaccine group responded well to all adjuvants tested for a humoral response in the Combo5 study. In the Combo5 study, the cellular immune response was enhanced with the use of the SMQ adjuvant over Alum, resulting in an increase of cytokines such as IL-6, IL-10, TNF-α and IFN-γ [15]. Therefore, the functionality of adjuvants in formulations should be better understood, including whether antigens are able to compete for adsorption. A sequential adsorption may be needed for the optimal association for all antigens.

Mac and Cpa, meanwhile, showed an increase in the IgG titre when M proteins were included in the formulations. This could be due to the synergistic effects of the other antigens in the combination formulation. and this correlated with the functional activity for Mac. We demonstrated that immune sera against Mac was able to block its cleavage of human IgG to a remarkable level and that this was evident in all the vaccine formulations. The “ALL” and “NoM” combinations showed higher titratable neutralising effects than the individual immunisations. In the confines of an OPA with mouse sera, the effect of Mac on human IgG cleavage would not be evident. The immune sera did show minor killing activity compared to the nonimmune sera (*p* < 0.1); however, this was significantly lower than the killing activity with M1-immune sera and comparable with MalE-immune sera, which were shown to have a negligible IgG response. There has been a dual activity of Mac reported, where it has been shown previously to also block the binding of Fc receptors, theorised in combination with IgG cleavage to prevent the opsonisation and phagocytosis of GAS cells [22]. The effect of Mac vaccination on the prevention of systemic infection would therefore be of interest for future studies. In a mouse model of infection, it has been shown to not be essential for virulence [34]; however, a lack of specificity of Mac to mouse IgG may, in part, account for this, and the use of humanised mice with human immunoglobulins may be a more suitable model to study the role of this virulence factor [35].

Cpa is a pilin tip protein, predicted to bind collagen, which plays a role in the adhesion to HaCaT cells [16,17]. Numerous serotypes have been found and show limited cross-reactivity. In this present study, we included Cpa as a representative of the surface-exposed colonisation factors with extracellular matrix (ECM) binding properties. The optimisation of binding conditions demonstrated that M1 Cpa can bind to collagen I, as previously reported [16], but additionally, can bind to fibronectin. The Cpa binding of fibronectin has not been previously reported experimentally. Cpa (Spy0125) from M1 shows structural and sequence homology to the *Streptococcus equi subsp. Equi*-secreted fibronectin-binding protein FNE [36]. The homology encompasses the region containing the domain structure in Cpa responsible for an internal thioester bond required for efficient host cell interactions [37,38]. FNE binds to the gelatin-binding domain of fibronectin, an alternative binding mechanism to the classic canonical repeat motifs found in common fibronectin-binding proteins. This may explain our observation of Cpa binding to fibronectin without containing classic binding repeats. In our study, incubation with both immune and nonimmune sera showed the inhibition of binding to fibronectin due to the intrinsic blocking activity of sera proteins; therefore, the IgGs were purified and investigated for the blocking of Cpa binding. Though this refinement improved the assay with nonimmune IgG, there was only a negligible reduction in the binding with immune IgGs in the confines of the current assay. SF370 (M1) GAS cells can bind HaCaT cells, but recombinant C-terminal Cpa could not in a previous study, despite immune sera against the Cpa-blocking binding of GAS cells [17]. Similarly, anti-Cpa sera blocked the HaCaT binding of M28 GAS [30]. This highlights that, for certain aspects of vaccine evaluations, the entire organism should be considered rather than the individual vaccine component, especially a component of a polymer structure. Despite this hypothesis, it would be of interest to investigate the effect of IgA blocking on activity, as mucosal immunity is more relevant to the prevention of colonisation in the nasopharynx. This would also help to establish whether the induction of systemic or mucosal immunoglobulins correlate with the blocking of colonisation, as immunogenic epitopes targeted by IgG may not be effective. A previous study of M6 binding to Detroit-562 pharyngeal cells demonstrated that only IgA was effective at blocking adherence, while IgG-blocked epitopes responsible for invasion phenotypes [39].

Recombinant MalE did not generate a high immune response following immunisation in the present study. In previous studies, it was highlighted as a key antigen due to the high level of antibodies present in IVIG [40], which we were able to reproduce in our analysis of IVIG. However, this was not reflected in our immunogenicity study where the antibody titres were not significantly above the pre-immune levels. Previous immunisations with MalE in our work demonstrated low but quantifiable IgG against MalE, as evidenced by Western blotting, but this was not replicated in the subsequent study. This may be related to the rate of adsorption to the adjuvant, leading to competition between antigens that affects the immune response, as seen with SpyCEP, and as such, improvements on the formulation should be investigated. The stimulation of mucosal immunity may demonstrate the improved presentation of MalE to the immune system and an increased humoral response, as observed with an influenza A virus vaccine that incorporated an IgA-inducing protein as an adjuvant for IgG and IgA responses [41].

It has become increasingly clear that, to target broad GAS disease presentation with global serotype prevalence, a multicomponent vaccine will be necessary. Several preclinical studies have been conducted for combination vaccines with encouraging protection data from various in vivo infection models [41]. These multicomponent vaccines can improve the cross-serotype and multi-disease protection needed for an infectious agent such as GAS. However, multicomponent vaccines have some limitations, such as the incompatibility of antigens with adjuvants, epitopic suppression and interactions between components that cause conformational changes [15]. Our data highlighted that, to ensure effective protection from each component, the immunogenicity from the formulation needs to be understood. This has previously been highlighted for *Haemophilus influenza* type B (Hib) vaccines, where the adsorption to adjuvant of the candidate antigens was hindered by other vaccine components [42]. Further works should investigate the adsorption kinetics of antigens to adjuvants individually and compare combination adsorptions together or in a stepwise approach. Encouragingly, the absence of M proteins showed no loss of immunogenicity for any antigens compared with the individual immunisations and resulted in killing activity in an OPA not significantly below that for M1 alone or the “ALL” formulation. This demonstrates that targeted antigen selection for inclusion can lead to comparable immunity to the M protein without compromising a specific antigen-neutralising activity. Further work is needed to understand the effective prevention of colonisation and survival in the nasopharynx from combination vaccines targeting multi-disease presentations of GAS and whether this also results in immunity from systemic infections. In vitro models are useful tools to reduce and refine the use of animals; however, the extrapolation of in vitro assays towards protective immunity will need to be validated with infection models. This was seen with *S. pyogenes* nuclease A (SpnA), where a robust IgG response and neutralising activity did not correlate with protection, despite a role for SpnA in pathogenesis [43].

## 5. Conclusions

To conclude, with multicomponent vaccines likely to be a viable long-term solution to prevent GAS globally, more understanding of the effects of the formulations is required to ensure an effective adjuvant adsorption and immune response against all candidate antigens. Despite the prevention of pharyngitis being the WHO-preferred product characteristic [2], this will not protect against systemic infections, so the incorporation of both colonisation factors and systemic virulence factors is of high importance. As an OPA is not indicative of functional immunity for several key candidate vaccine antigens, more assays will need to be incorporated into studies to understand the correlates of protection. Finally, a better understanding of the mucosal response to infection and immunisation is needed, particularly in consideration of the prevention of colonisation and pharyngitis, to reflect the WHO-preferred product characteristics of a vaccine.

## Figures and Tables

**Table 1:** Analysis of the selected protein antigens for study inclusion.

Antigen (SF370)	Function	Location	Colonisation	Invasion	Immune Evasion	Vaccine	Titre in IVIG	Ref.
M protein (*emm1*, SPy_2018)	Main virulence factor	Cell wall				30 valent J8 StreptinCor	24,628 (M1) 17,392 (M3)	[28]
SpyCEP (SPy_0416)	IL-8 protease	Cell wall/secreted				GSK combo, Combo5	6038	[28]
Mac (IdeS) (SPy_0861)	IgG protease	Secreted				-	12,203	[22,29]
Cpa (AP1) (SPy_0125)	Pilin tip	Cell wall				*L. lactis*	3329	[17,30]
MalE (SPy_1294)	Maltodextrin binding, saliva survival	Membrane				-	828	[19,31]

Five protein antigens were selected for inclusion in the present study. The functional role and cellular location of each antigen is listed. Grey boxes indicate involvement in the stages of infection. -, not previously included in any preclinical or clinical vaccine studies. Titres are presented as a dilution yielding a signal of OD 0.5 at 450 nm by ELISA.

**Table 2:** IgG response against each antigen in the individual and combination immunisations.

Group	Geometric Mean Titre of IgG Response against Individual Antigens (95% CI)					
	M1	M3	SpyCEP	Mac	Cpa	MalE
Pre-immune	1	1	11	1	1	88
Individual	34,542 (16,943–70,422)	97,715 (38,142–250,333)	125,934 (57,608–275,299)	4856 (1945–12,127)	2870 (188–43,844)	93 (34–258)
Combination “ALL”	29,503 (20,367–42,736)	60,579 (33,142–110,729)	31,881 ** (16,146–62,950)	10,549 * (7333–15,175)	51,741 *** (39,617–67,575)	268 * (174–411)
“NoM”	155 (94–257)	11 (1–101)	115,946 ^++^ (67,399–199,462)	12,208 (7287–20,453)	11,800 ^+^ (3529–39,458)	NT

“ALL”, the combination with all antigens, and “NoM”, the combination of SpyCEP, Mac, Cpa and MalE. NT, not tested. Statistical analysis by the Mann–Whitney *U* test: * *p* < 0.05, ** *p* < 0.01 and *** *p* < 0.001, comparisons of the individual to combinations “ALL” or “NoM”. ^+^ *p* < 0.05 and ^++^ *p* < 0.01, the comparison of “NoM” to combination “ALL”.

## Data Availability

Not applicable.

## Supplementary Materials

The following are available online at https://www.mdpi.com/article/10.3390/vaccines9091025/s1: Figure S1: Schematics of the GAS protein antigens, Figure S2: Vaccine antigens produce antibodies that can recognize native and recombinant proteins and Figure S3: Neutralising antibodies can be titrated.
