# Supplementary figures and images for: Multicomponent Vaccines against Group A Streptococcus Can Effectively Target Broad Disease Presentations
Source: Vaccines (Basel). 2021 Sep 15;9(9):1025. doi: 10.3390/vaccines9091025 (PMC8473114; doi:10.3390/vaccines9091025)

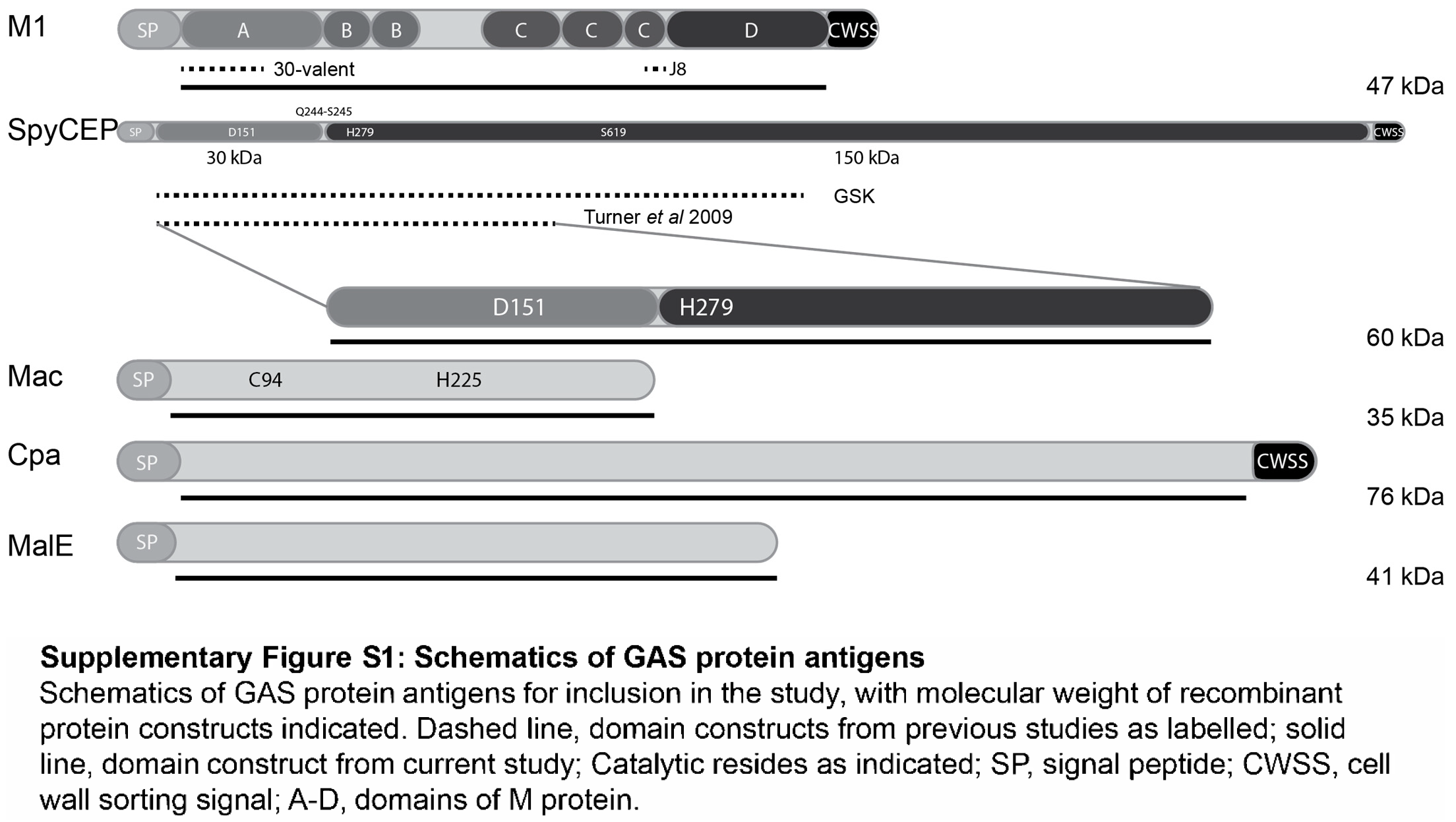

Supplement: Supplementary file 1 [file vaccines-09-01025-s001.zip › Supplementary Figure S1.png]

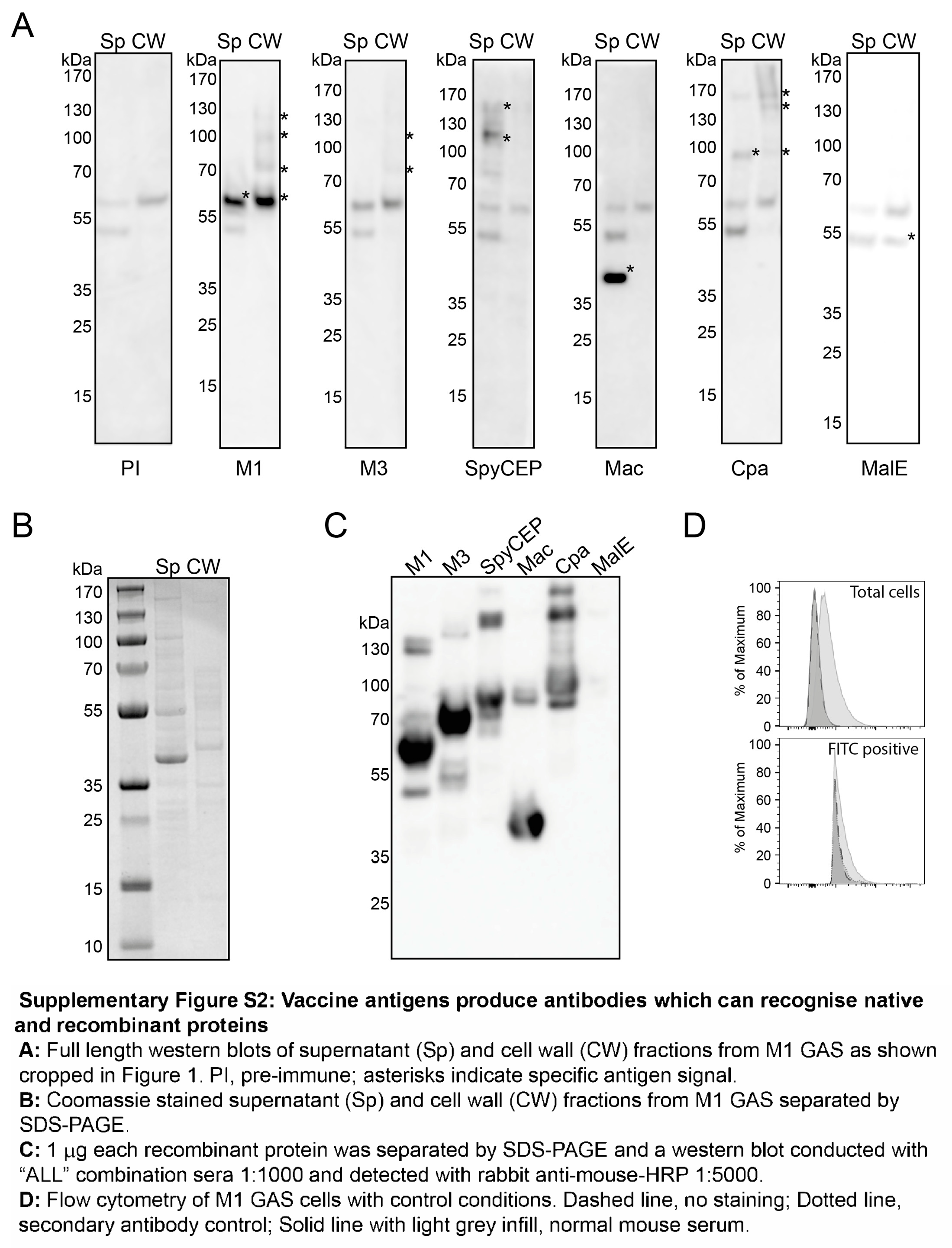

Supplement: Supplementary file 1 [file vaccines-09-01025-s001.zip › Supplementary Figure S2.png]

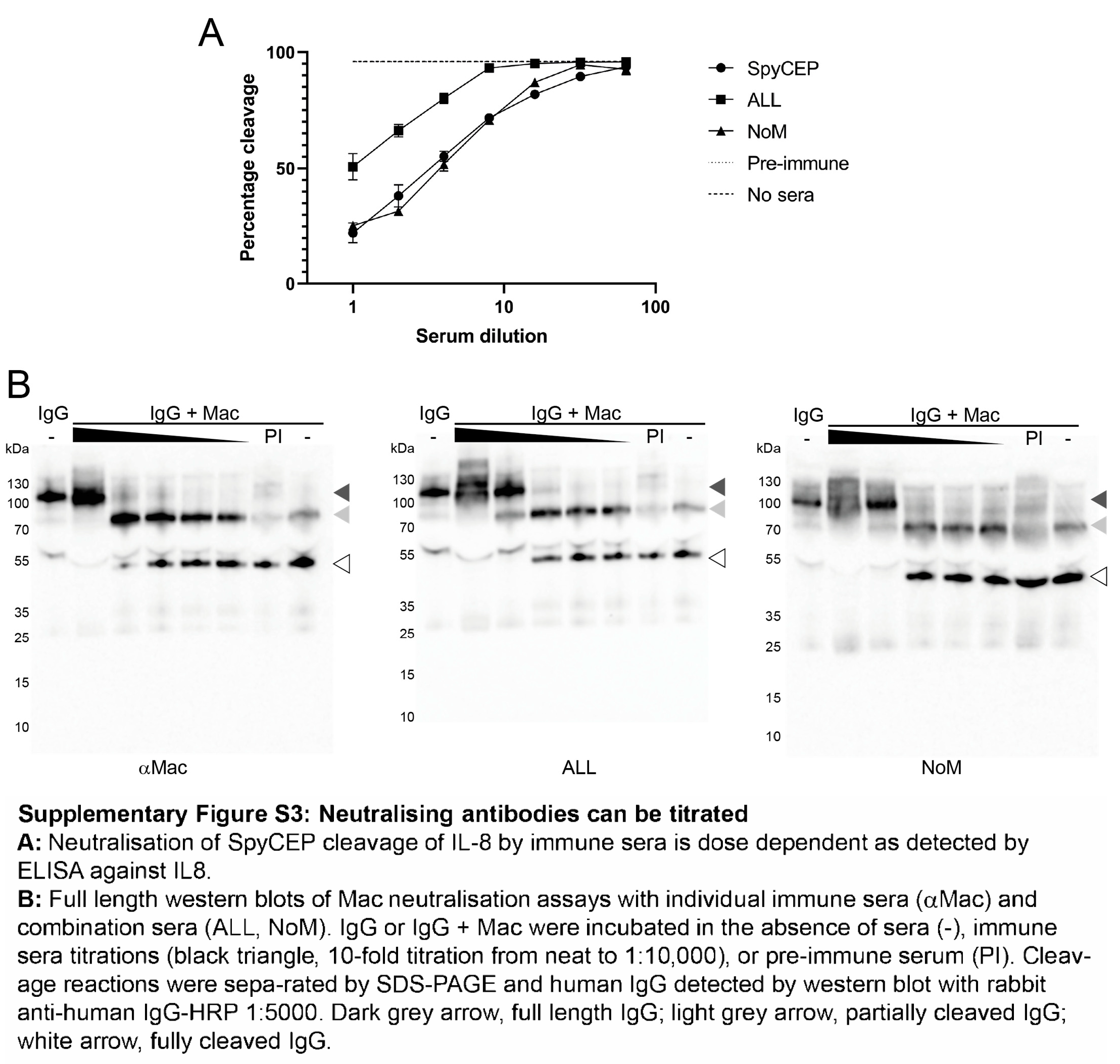

Supplement: Supplementary file 1 [file vaccines-09-01025-s001.zip › Supplementary Figure S3.png]
